# Description of Referrals for Colposcopy in a Hospital in Brazil

**DOI:** 10.1055/s-0040-1708886

**Published:** 2020-03

**Authors:** Stephanie Hein de Carvalho, Dayana Aparecida Nacimento Rosa, André Luis Ferreira Santos, Andréa Paula Peneluppi de Medeiros

**Affiliations:** 1 Department of Gynecology, Universidade de Taubaté, Taubaté, SP, Brazil

**Keywords:** colposcopy, neoplasms of the cervix, prevention, histology, colposcopia, neoplasias do colo uterino, prevenção, histologia

## Abstract

**Objective** To describe the referral for colposcopy in a Hospital in Brazil and the relative frequency of patients who benefited from it, considering the correct indications for the examination and its final diagnoses.

**Methods** A retrospective study was performed in the colposcopy service database of the Hospital Universitário de Taubaté, Taubaté, state of São Paulo, Brazil. The frequency validated in the analysis of the medical records of women referred for clinical indication or cytological alteration, attended from March 2015 to March 2017. The population selected and analyzed included 256 results that were correlated to the cytological, clinical data and the result of the colposcopy.

**Results** Of the women referred, 45% presented out of the age of screening according to the guidelines of cervical cancer screening, 8.6% being adolescents and young adults < 25 years old, and 36.4% of the patients being ≥ 65 years old. A total of 50% of the patients had no indication of colposcopy, that is, normal cytologies, benign changes, ectopia, cervicitis, atypical squamous cells of indeterminate significance (ASC-US) and low-grade intraepithelial lesion (LSIL) without persistence and normal clinical appearance. A total of 39.84% who underwent colposcopy had high-grade lesion or cancer results, thus benefiting from the adequate referral.

**Conclusion** Most (60.16%) of the patients referred to the colposcopy service did not benefit from the referral for results without changes, such as negative colposcopies, histologies with no cervical intraepithelial neoplasm (CIN) or only CIN 1, or were out of the age for screening. These findings therefore demonstrate a significant number of unnecessary and inadequate referrals.

## Resumo

**Objetivo** Descrever os encaminhamentos para colposcopia em um hospital no Brasil e a frequência relativa dos pacientes que foram beneficiados, considerando as indicações corretas para o exame e seus diagnósticos finais.

**Métodos** Foi realizado um estudo retrospectivo no banco de dados do serviço de colposcopia do Hospital Universitário de Taubaté, Taubaté, SP, Brasil. A frequência validou em análise de prontuários de mulheres encaminhadas por indicação clínica ou alteração citológica, atendidas no período de março de 2015 a março de 2017. A população selecionada e analisada incluiu 256 resultados que estavam correlacionadas aos dados citológicos, clínicos e com o resultado da colposcopia.

**Resultados** Das mulheres encaminhadas, 45% apresentaram-se fora da idade de rastreamento conforme as diretrizes de rastreio de câncer de colo uterino, sendo que 8,6% eram adolescentes e adultas jovens < 25 anos de idade e 36,4% das pacientes tinham idade ≥ 65 anos. Um total de 50% das pacientes não possuía indicação de colposcopia, ou seja, citologias normais, de alterações benignas, ectopia, cervicite, células escamosas atípicas de significado indeterminado (ASC-US) e lesão intraepitelial escamosa de baixo grau (LSIL) sem persistência e aspecto clínico normal. Um total de 39,84% das pacientes que realizaram a colposcopia tiveram resultados de lesão de alto grau ou câncer e, dessa forma, se beneficiaram com o encaminhamento adequado.

**Conclusão** A maioria (60,16%) das pacientes encaminhadas para o serviço de colposcopia não se beneficiou com o encaminhamento, por resultados sem alterações como colposcopias negativas, histologias com ausência de neoplasia intraepitelial cervical (NIC) ou apenas NIC 1, ou estavam fora da idade de rastreamento. Esses achados demonstram, portanto, significativo número de encaminhamentos desnecessários e inadequados.

## Introduction

Recognition of preinvasive phases of cervical cancer is fundamental and effective, as it allows reducing the incidence and mortality.^1^ ^2^ ^3^ ^4^ ^5^ Cervical intraepithelial neoplasms (CINs) are considered precursor lesions of squamous cancer of the uterine cervix. They are divided into grades 1, 2 and 3, both by the morphological aspect of the epithelium compromise and also by its variable evolutionary potential.^6^

The oncologic cytology (OC), also known as Pap smear test,^7^ when performed within the quality standards, presents a coverage of 80% for invasive cancer and, if the initial lesions are treated, the reduction in the rate of invasive cervical cancer can reach 90%.^8^ ^9^ ^10^ Colposcopy is rightly indicated when there is alteration in the screening cytology. Recent papers show that when you associate cytology results with colposcopy, the sensitivity increased to 98%. The main role of it is to determine the site of the biopsy that provides the histological diagnosis, which is the gold standard and the basis of the treatment.^11^

Due to this scenario, the need to improve the secondary prevention of cervical cancer was evidenced. In view of the epidemiological situation of cervical cancer in Brazil and the difficulties of vacancies in the referral services of colposcopy, the present study aimed to describe the frequency of women being adequately forwarded to the referral service and who benefited from it, according to the correct indications for colposcopic examination and the final histological diagnoses of high grade intraepithelial lesion (HSIL) and cancer.

## Methods

This is a retrospective study describing the referral for colposcopy in a Hospital in Brazil. The study was conducted at the Hospital Universitário de Taubaté, Taubaté, state of São Paulo, Brazil. A total of 329 medical records of women who were attended at the service were evaluated through the referral to the colposcopy clinic, but the population selected resulted in 256 women because they were the only ones who had cytological and clinical data with colposcopy results, which were needed for the study. The following results were classified as negative: benign alterations, cervicitis, CIN 1, and normal; and as positive, HSIL and cancer. The classification of altered clinic was given to referrals made because of alterations in the cervical clinic and/or after related procedures.

The exclusion criteria were patients who were referred for vulvar lesions, leukorrhea, vaginal pruritus, postcoital bleeding, dyspareunia, endometrial hyperplasia, hysterectomy due to myomas, ovarian cysts, other alterations that do not represent cases of cervical cancer prevention and cases where colposcopy was not necessary. Also, benign cytological alterations and patients < 25 years old with HIV were excluded.

During the analysis of the histology results, it was found that there was a reduction in the number of participants to 218. This is due to the fact that some patients did not return to verify the results of the biopsy performed and to those with normal colposcopy, when a biopsy was not necessary.

A survey was made of the electronic medical records of all patients referred to the colposcopy service in the period of March 2015 to March 2017. We have only used the data already registered in the electronic records. We did not submit patients to any procedure or summonations and kept the anonymity of the women. Because it is a school/university hospital, in the first care, the patient is asked to consent to the use of her medical data, recorded for academic purposes. Thus, the ethics Committee extended this consent to the research performed at the institution.

The research was approved by the Ethics and Research Committee of the Universidade de Taubaté (Number: 2.285.907 CAAE: 74646517.6.0000.5501). The ethical aspects of the research involving human beings were respected, according to the recommendations of resolution No. 196/96.

The data collection form was composed by data such as: identification of patients regarding age, the reason for referral to the service, the cytological diagnosis (made by the laboratory contracted by the city hall [ACTA] and private ones), the colposcopic diagnosis, biopsy and, finally, the relevant observations in the management. The colposcopy and biopsies were made by medical doctors from the hospital, most of them teachers of the Universidade de Taubaté. All of the results (histology) of the biopsies were given by the ACTA laboratory.

The data was collected from the hospital information system registered in electronic media and all of the variables involved in the present study were stored in Excel for MAC version 16.17 (Microsoft Corporation, Redmond, WA, USA). The data was analyzed by IBM SPSS Statistic for Windows, Version 24.0 (IBM Corp., Armonk, NY, USA). The results went through a review, were checked and presented in contingency tables and discussed according to the pertinent literature. A descriptive analysis of the collected data was performed by presenting the simple frequency (absolute numbers and percentages).

## Results

Out of the 256 patients attended at the colposcopy clinic, 22 (8.6%) were < 25 years old, 242 (94.5%) presented cytopathological reports (with and without alterations), and 218 (85.1%) were submitted to colposcopy-guided biopsy, obtaining histological results. The mean age of the patients was 42.3 years old, and the age range was from 15 to 91 years old. With highest representativeness was the age group between 45 and 54 years old, corresponding to 30.2% of the total number of patients. Out of the total of 256 patients, 128 (50%) had no indication of colposcopy (< 25 years old, cytologies of benign alterations, cervicitis, atypical squamous cells of indeterminate significance [ASC-US] and low-grade intraepithelial lesion [LSIL] without persistence, and normal clinical aspect). In Table 1, it is verified that 38 patients (14.9%) had no need to perform biopsy because they presented normal colposcopy.

**Table 1:** Prevalence of high-grade lesions in patients undergoing colposcopy, correlating cytological and clinical diagnoses with colposcopic and histological diagnosis of 256 patients attended at a colposcopy clinic

Cytology	Normal Colposcopy		Altered Colposcopy				Total	
			Negative		Positive			
	*n*	%	*n*	%	*n*	%	*n*	%
AGC	01	0.4	05	2.0	01	0.4	07	2.8
ASC-US	05	2.0	36	14	12	4.7	53	20.7
ASC-H	0	0	10	3.9	10	3.9	20	7.8
LSIL	13	5.1	33	12.9	18	7.0	64	25
HSIL	01	0.4	08	3.1	61	23.8	70	27.3
Negative	16	6.2	12	4.7	0	0	28	10.9
Alt Clinical	02	0.8	12	4.7	0	0	14	5.5
Total	38	14.9	116	45.3	102	39.8	256	100

Abbreviations: AGC, atypical glandular cells; AIS, adenocarcinoma in situ; Alt Clinical, altered clinic; ASC-H, intraepithelial lesion with a tendency to high degree; ASC-US, atypical squamous cells of indeterminate significance; HSIL, high-grade intraepithelial lesion; Invasive CA, invasive carcinoma; LSIL, low-grade intraepithelial lesion; Negative, benign and cervicitis alterations.

The cytological and clinical data were analyzed with the results of the colposcopy, being classified as “negative” information the results of benign alterations, cervicitis, CIN 1 and normal colposcopy; and as “positive” information the HSILs and cancer. The altered clinical classification (Alt Clinical), refers to clinical alteration of the colon or after uterine cervix procedure (Table 1).

Out of the 256 patients who underwent colposcopy, we obtained 38 cases (14.9%) with normal results, 116 cases (45.3%) of negative colposcopy for high-grade lesions and 102 cases (39.8%) positive for high-grade injury. When comparing the cytological and colposcopic results, we found that of the 7 cases of atypical glandular cells (AGC), only 1 case was positive for high-grade lesions (14.3%), with another 5 (71.4%) negative results, and 1 case with normal colposcopy. Of the 53 cases of ASC-US there were 12 cases (22.6%) with positive colposcopy, 36 cases (67.9%) with negative colposcopy and 5 normal cases (9.4%). Out of the 20 intraepithelial lesion with a tendency to high degree (ASC-H) cytologies, all had altered colposcopy, being half with negative colposcopy and half with positive colposcopy for high-grade lesions. Of the 64 cases of LSIL, 13 cases (20.3%) had normal colposcopy, 33 cases (51.5%) were negative and 18 cases (28.1%) were positive. Out of the 70 HSIL results, only 1 case (1.4%) had normal colposcopy, 8 cases (11.4%) were negative and the majority (87.1%) had positive colposcopy for HSILs. There were no positive colposcopic results of negative cytologies, with 12 cases (42.8%) of the 28 negative results and 16 cases (57.1%) with normal colposcopy. Regarding the referrals due to clinical alterations, out of the 14 cases, there were no cases with positive colposcopy, 12 cases (85.7%) were negative and only 2 cases (14.3%) presented normal colposcopy (Table 1).

In the 218 patients who underwent biopsy, the histological results showed: 86 cases (39.5%) had no CIN results, 30 cases (13.7%) CIN 1, 93 cases (42.6%) were diagnosed as CIN ⅔, 4 cases (1.9%) of adenocarcinoma in situ and 5 cases (2.3%) were considered as invasive malignant neoplasia (squamous carcinoma or adenocarcinoma). The highest incidence of CIN 1 was found in the age groups from 25 to 34 years old and between 45 and 54 years old, with 9 cases (4.1%) in each group. In relation to the CIN ⅔ result, the range between 25 and 34 years old presented a higher quantity, with 34 patients, corresponding to 15.6%. Regarding cancer (adenocarcinoma in situ [AIS] and invasive), the group of women between 45 and 54 years old presented the majority of cases (*n* = 4; 1.9%). Patients who had results of HSIL and cancer totaled 102 cases (46.8%). (Table 2).

**Table 2:** Distribution of histological diagnoses according to the age group of patients assisted at a colposcopy clinic

Age (years old)	Absence of CIN		CIN 1		CIN 2/3		AIS		Invasive CA		Total	
	*n*	%	*n*	%	*n*	%	*n*	%	*n*	%	*n*	%
< 25	09	4.1	06	2.8	02	0.9	0	0	0	0	17	7.8
25–34	08	3.7	09	4.1	34	15.6	0	0	0	0	51	23.4
35–44	24	11	04	1.8	23	10.5	01	0.5	0	0	52	23.8
45–54	31	14.2	09	4.1	22	10	01	0.5	03	1.4	66	30.2
55–64	06	2.8	0	0	06	2.8	0	0	0	0	12	5.6
≥ 65	08	3.7	02	0.9	06	2.8	02	0.9	02	0.9	20	9.2
Total	86	39.5	30	13.7	93	42.6	04	1.9	05	2.3	218	100

Abbreviations: AIS, adenocarcinoma in situ; CIN, cervical intraepithelial neoplasm; Invasive CA, invasive carcinoma.

Information of the cytologies were analyzed with the histological results, independently of the age group, totaling the same 218 results. Of this total, 116 (53.2%) of the biopsied cases showed negative histological results for a HSIL. Of the 6 reports that presented cytopathological findings suggestive of AGC, histopathology revealed one (16.6%) case of CIN ⅔ and 5 (83.3%) negative cases (no injuries). Of the 48 cases of ASC-US, 12 (25%) cases corresponded to CIN 1, 10 (20.8%) cases of CIN ⅔, 2 (4.1%) cases of invasive adenocarcinoma and 24 (50%) negative cases for CIN. Of the 20 ASC-H cytologies, 10 cases (50%) had no lesion and the other half had CIN ⅔. In the 51 cases of LSIL cytology, we found 17 (33.3%) negative cases, 16 cases (31.4%) CIN 1, 17 (33.3%) CIN ⅔ and 1 (1.9%) case of adenocarcinoma in situ. In 69 HSIL cytologies, we had 7 (10.1%) negative cases, 1 (1.44%) case of CIN 1, 55 (79.7%) of CIN ⅔, 3 (4.34%) cases of AIS and 3 cases of invasive adenocarcinoma. Of the 12 negative cytologies for CIN, 11 (91.7%) were confirmed negative histologies and there was 1 (8.3%) case of CIN 1. In the 12 cases of altered clinical, all obtained negative results for “positive” information, that is, CIN 2, AIS and invasive carcinoma (CA) histology (Table 3).

**Table 3:** Correlation between cytology and histopathology of 218 patients assisted at a colposcopy clinic

Cytology	Histopathology											
	Negative		CIN 1		CIN 2/3		AIS		Invasive CA		Total	
	*n*	%	*n*	%	*n*	%	*n*	%	*n*	%	*n*	%
AGC	05	2.3	0	0	01	0.5	0	0	0	0	06	2.8
ASC-US	24	11	12	5.5	10	4.6	0	0	02	0.9	48	22
ASC-H	10	4.6	0	0	10	4.6	0	0	0	0	20	9.2
LSIL	17	7.8	16	7.3	17	7.8	01	0.5	0	0	51	23.4
HSIL	07	3.2	01	0.5	55	25.2	03	1.4	03	1.4	69	31.7
Negative	11	5.0	01	0.5	0	0	0	0	0	0	12	5.5
**Alt Clinical**	12	5.5	0	0	0	0	0	0	0	0	12	5.5
Total	86	39.4	30	13.8	93	42.7	04	1.9	05	2.3	218	100

Abbreviations: AGC, atypical glandular cells; AIS, adenocarcinoma in situ; Alt Clinical, altered clinic; ASC-H, intraepithelial lesion with a tendency to high degree; ASC-US, atypical squamous cells of indeterminate significance; HSIL, high-grade intraepithelial lesion; Invasive CA, invasive carcinoma; LSIL, low-grade intraepithelial lesion; Negative, benign and cervicitis alterations.

Using the histological results from colpodirected biopsies as the gold-standard test, a specificity of 77.8% and a sensitivity of 70.5% were obtained for cytology. The positive predictive value (PPV) was 75.7% and the negative predictive value (NPV) was 72.9% for precursor lesions and cancer.

## Discussion

It was found that 102 patients (39.84%) of the 256 patients who underwent colposcopy had results of HSIL or cancer, meaning they benefited from adequate referral. The remaining 60.16%, with negative takeoff results and histologies with absence of CIN or only CIN 1, demonstrate a large amount of inadequate referrals.

The most altered cytological findings were HSIL, followed by LSIL and ASC-US, corresponding to 70 (27.3%), 64 (25%) and 53 (20.7%) forwarded cases, respectively, followed by intraepithelial lesion with a tendency to high degree (ASC-H) cytologies (7.8%) and ACG (2.7%). These results are similar to those observed in the project^12^ made for the standardization of the terminology of HPV- Lower Anogenital Squamous associated lesions, in which they found the alterations LSIL, HSIL and high-grade squamous intraepithelial lesions not excluding epidermoid carcinoma (HSIL/CA) in higher frequency, corresponding to 99.1% of the total lesions.^13^ ^14^ ^15^

A sensitivity of 70.5% and specificity of 77.8% was found for cytology, demonstrating its value as a screening test for these types of lesions. The PPV of 75.7% is in line with what was observed in the literature searched in a cross-sectional observational study^16^ performed in a Women Health Center in the state of Piauí, Brazil, with a PPV index for cytology ranging from 62.2% to 85%.

Between cytology and histopathological results, from the colpodirected biopsy, there was a significant concordance of 91.7% of the negative cases and of 79.7% for HSIL. There was a divergence in the cytological result of ASC-US, in which ∼ 20.8% of the cases corresponded to high-grade precursor lesions, in addition to two cases of invasive adenocarcinoma. This result is significant given that in the guidelines of the Ministry of Health (MH)^2^ the cytological diagnosis of ASC-US corresponds to the absence of lesions in the vast majority of cases, not being a formal indication for colposcopy, diverging from our findings, and may infer quality problems in the cytological examination in our region.

Out of the 20 ASC-H cytologies, 10 cases (50%) had no lesion and the other half had CIN ⅔. These findings are in line with the data obtained in the study by Joseph et al,^14^ where all surgical pathology reports of cervical colposcopic biopsies, cone biopsies, and hysterectomy specimens of 772 consecutive patients were correlated with cytology results, in which ASC-H cases there was a wide alteration in histopathology, in addition to finding a cancer occurrence above the expected. According to the Carvalho et al,^16^ who made a comparative evaluation of the positive cytology, colposcopy and histopathology, this evidences a tendency of cytology to underestimate the lesions, since we consider histology as the gold standard. This can occur due to errors in the sampling, collection and interpretation of the material. Among the diagnosed lesions, there was a higher prevalence of CIN ⅔ (42.7%).

Precisely because the technique of performing the exam is subject to errors, physicians and nurses who collect the samples should be well-trained, specially pathologists who through their reports determine the indication of the colposcopy itself or may induce iatrogenic procedures. It is also important to remember that the results obtained have relevance in the indices of the Ministry of Health, impacting the public policies.

It is necessary to emphasize as a limitation of the analysis of the present study the fact that there is usually a long wait time for colposcopy or biopsy. Thus, the lesions are subjected to regression or progression, which may be distally from the results obtained in the cytology.

The age group between 25 and 34 years old contains the highest number of high-grade lesions (CIN ⅔), corresponding to 15.6%, and the patients between 45 and 54 years old with a higher rate of cancer outcomes (1.9%). A total of 4 cases of AIS were found in the study, and 5 cases of invasive malignant neoplasia; 4 (44.4%) of these lesions were found in patients aged between 45 and 54 years old and 4 in (36.4%) patients aged ≥ 65 years old. In the literature,^16^ similar results were found, observing higher risk before 64 years old and falling after 65 years old. From the 22 (8.6%) adolescents and young adults < 25 years old, 17 (7.8%) underwent biopsy, and only 2 cases of HSIL were confirmed by histology, corresponding to 0.9% of the total cases. Except for these two cases, all of the other patients in this group underwent cytology and were referred to colposcopy unnecessarily.

These results corroborate the proposal of the Ministry of Health that the cytological examination should be offered to women in the age group of 25 to 64 years old and who have had sexual activity.^6^ This age-group priority as the target population is justified by the higher occurrence of high-grade lesions, which can be effectively treated so that they do not evolve to cancer. According to the World Health Organization (WHO), the incidence of this cancer increases in women between 30 and 39 years old and reaches its maximum in the 5^th^ or 6^th^ decade of life. Before the age of 25 years old, HPV infections and low-grade lesions prevail, which in the vast majority of cases will regress spontaneously and, therefore, can only be monitored according to clinical recommendations. After 65 years old, on the other hand, if the woman has performed the preventive exams regularly, with normal results, the risk of developing cervical cancer is reduced due to its slow evolution. That was observed in an article^4^ based on the history of the use of colposcopy in the United States and its possibility as a cervical cancer control tool.

Considering that there are several long-term stages for progression, so that the precursor lesions of the cervix become a cancer (natural history of the disease), screening with early detection allows adequate treatment and cure in most cases.

Being a retrospective study and having few publications with an objective similar to our own, we find the limitations of the present study: the nonrealization of the calculation of association measures and the evaluation of statistical significance. However, as this is a scientific initiation work, it was prioritized as an objective to establish, at first, the profile of this aggravation in the target population and to evaluate the indications of colposcopy in the public system. The strong point of the study was to detect the need for continuing medical education with the primary care professionals to homogenize, standardize and follow the guidelines for better optimization of services.

## Conclusion

The results of the present study ratify the importance of cytology as a screening method, colposcopy and histopathological examination as diagnostic confirmation and of assessing the need for adequate treatment in patients with HSIL and cancer. In the present study, 102 (39.8%) patients benefited with a final diagnosis of HSIL. There was a large amount of unnecessary referrals, burdening the health system and occupying vacancies of patients who really need it. In the present paper, we were concerned with the cytological diagnosis of ASC-US of referrals, since 20.8% of the cases corresponded to high-grade precursor lesions, in addition to two cases of invasive adenocarcinoma. This result is of relevance because, in the guidelines of the MH^2^, the cytological diagnosis of ASC-US corresponds to the absence of lesions in the vast majority of the cases, not being a formal indication of colposcopy, diverging from our findings, inferring quality problems in the cytological examination in our region. It is also confirmed that the concordance of the methods used in the diagnosis of cervical lesions is important in the therapeutic approach, when we consider that false positive results generate unnecessary costs and may induce an iatrogeny. These findings invite the reflection on the importance of health education programs, medical training and other primary care professionals regarding the prevention of cervical cancer, with a view to quaternary prevention. In this way, they would increase the benefits of referral to the population that really needs colposcopy.
